# Construction and validation of a readmission risk prediction model for elderly patients with coronary heart disease

**DOI:** 10.3389/fcvm.2024.1497916

**Published:** 2024-12-18

**Authors:** Hanyu Luo, Benlong Wang, Rui Cao, Jun Feng

**Affiliations:** Department of Cardiology of Lu'an People's Hospital, Lu'an Hospital of Anhui Medical University, Lu'an, China

**Keywords:** coronary heart disease, readmission, prediction model, XGBoost, TyG-BMI

## Abstract

**Background:**

To investigate the risk factors for readmission of elderly patients with coronary artery disease, and to construct and validate a predictive model for readmission risk of elderly patients with coronary artery disease within 3 years by applying machine learning method.

**Methods:**

We selected 575 elderly patients with CHD admitted to the Affiliated Lu’an Hospital of Anhui Medical University from January 2020 to January 2023. Based on whether patients were readmitted within 3 years, they were divided into two groups: those readmitted within 3 years (215 patients) and those not readmitted within 3 years (360 patients). Lasso regression and multivariate logistic regression were used to compare the predictive value of these models. XGBoost, LR, RF, KNN and DT algorithms were used to build prediction models for readmission risk. ROC curves and calibration plots were used to evaluate the prediction performance of the model. For external validation, 143 patients who were admitted between February and June 2023 from a different associated hospital in Lu'an City were also used.

**Results:**

The XGBoost model demonstrated the most accurate prediction performance out of the five machine learning techniques. Diabetes, Red blood cell distribution width (RDW), and Triglyceride glucose-body mass index (TyG-BMI), as determined by Lasso regression and multivariate logistic regression. Calibration plot analysis demonstrated that the XGBoost model maintained strong calibration performance across both training and testing datasets, with calibration curves closely aligning with the ideal curve. This alignment signifies a high level of concordance between predicted probabilities and observed event rates. Additionally, decision curve analysis highlighted that both decision trees and XGBoost models achieved higher net benefits within the majority of threshold ranges, emphasizing their significant potential in clinical decision-making processes. The XGBoost model's area under the ROC curve (AUC) reached 0.903, while the external validation dataset yielded an AUC of 0.891, further validating the model's predictive accuracy and its ability to generalize across different datasets.

**Conclusion:**

TyG-BMI, RDW, and diabetes mellitus at the time of admission are the factors affecting readmission of elderly patients with coronary artery disease, and the model constructed based on the XGBoost algorithm for readmission risk prediction has good predictive efficacy, which can provide guidance for identifying high-risk patients and timely intervention strategies.

## 1 Introduction

One of the most prevalent cardiovascular conditions affecting the elderly is coronary heart disease (CHD). Primarily caused by coronary artery atherosclerosis, this condition leads to vascular blockages that result in myocardial ischemia and hypoxia, often referred to as “ischemic heart disease.” CHD imposes significant health risks and contributes to numerous fatalities. The global economic burden of CHD is substantial, with nearly 7 million deaths and 129 million disability-adjusted life years (DALYs) attributed to it each year. There is a marked variation in the mortality and incidence rates of CHD across different nations and regions. In developed countries, the incidence of CHD has seen a continuous decline over the past decades, potentially attributed to effective acute-phase treatments and enhanced primary and secondary prevention strategies. Conversely, in developing countries, the incidence of CHD exhibits considerable fluctuation, and the spread of Western dietary habits along with increasing sedentary lifestyles are expected to significantly drive the ongoing increase in CHD incidence in these regions (1). With the escalating trend of population aging, the incidence of CHD continues to rise. Notably, advanced age emerges as a prominent risk factor for cardiovascular diseases. Previous studies have demonstrated a higher prevalence of CHD among men aged over 40, with incidence rates soaring to 27.8% among individuals aged over 609 (2). Older adults are predisposed to aggregating multiple risk factors compared to their younger counterparts. These factors encompass high blood pressure, hyperlipidemia, diabetes, prolonged periods of sitting, and irregular medication schedules. Such a combination of risk factors can exacerbate disease advancement and elevate the likelihood of re-hospitalization (3). Repeated re-hospitalizations impose significant burdens and distress on both patients and their families, while also exerting considerable strain on patients' physical and mental well-being and overall quality of life. The increasing number of instances of coronary heart disease among China's senior population highlights the critical need for prompt intervention and assistance for those who are at risk. Reducing the number of readmissions may diminish the burden on families and society and improve the quantity and scope of life for senior citizens with coronary heart disease (4).

In recent years, numerous scholars have observed the promising outcomes of disease prediction and diagnosis achieved through the exploration of new artificial intelligence-based diagnostic models. For instance, Cao J (5) spotted the harmful effects of CHD and the need of early identification and used machine learning algorithms to create a risk model for the illness in young and middle-aged people. The study found that the XGBoost model was the best algorithm for predicting the likelihood of CHD in this population, providing an additional diagnostic strategy to reduce the risk of coronary heart disease in individuals who are young and middle-aged. A study conducted by Xiao Li (6), utilizing community-based physical examination data, investigated the CHD risk assessment model among elderly individuals. According to the study, the CHD risk assessment model that was created with the help of the XGBoost and logistic algorithms showed high levels of stability. This model serves as a valuable methodological reference for assessing CHD incidence risk within communities. Meng Qi et al. (7) wrote with the aim of developing and validating a nomogram model for identifying risk factors for CHD in a population with type 2 diabetes mellitus (T2DM) in northwestern China. The study identified independent risk factors associated with the development of CHD, including age, gender, hypertension, glycosylated hemoglobin, high-density lipoprotein cholesterol, low-density lipoprotein cholesterol, and Uyghur ethnicity, by analyzing data from 2118 T2DM patients. The results of the study showed that the developed nomogram showed good discrimination and calibration in both the training and validation sets, providing an effective clinical tool for predicting the risk of CHD in patients with T2DM. Zhao Jia et al. (8) developed and validated a new model for predicting the risk of CHD in snoring hypertensive patients with concomitant hyperhomocysteinemia in this study. The study developed a predictive model by analyzing relevant data and validated it to assess its accuracy and utility in predicting CHD risk. Wang Min et al. (9) conducted this retrospective observational study to develop and validate a CHD risk prediction model for snoring hypertensive patients. The study developed a risk prediction model by analyzing clinical data from patients and validated it to assess its effectiveness in predicting CHD risk. These studies provide new tools for coronary heart disease risk assessment in patients with hypertension and related disorders, and help clinicians better identify and manage cardiovascular risk in these patients. Machine learning is a method capable of making predictions and decisions by discerning patterns and rules from data. Through the construction of various methodologies and subsequent evaluation and comparison, the efficacy of disease prediction can be enhanced (10, 11). Currently, there has been a lack of comprehensive studies on readmission risk prediction models specifically tailored to elderly individuals with CHD in China. Therefore, this study utilizes modern machine learning methods to establish a predictive model for the risk of readmission among this specific population. This initiative aims to assist clinicians in offering supplementary diagnostic methods.

## 2 Materials and methods

### 2.1 Study population

**Figure 1:**
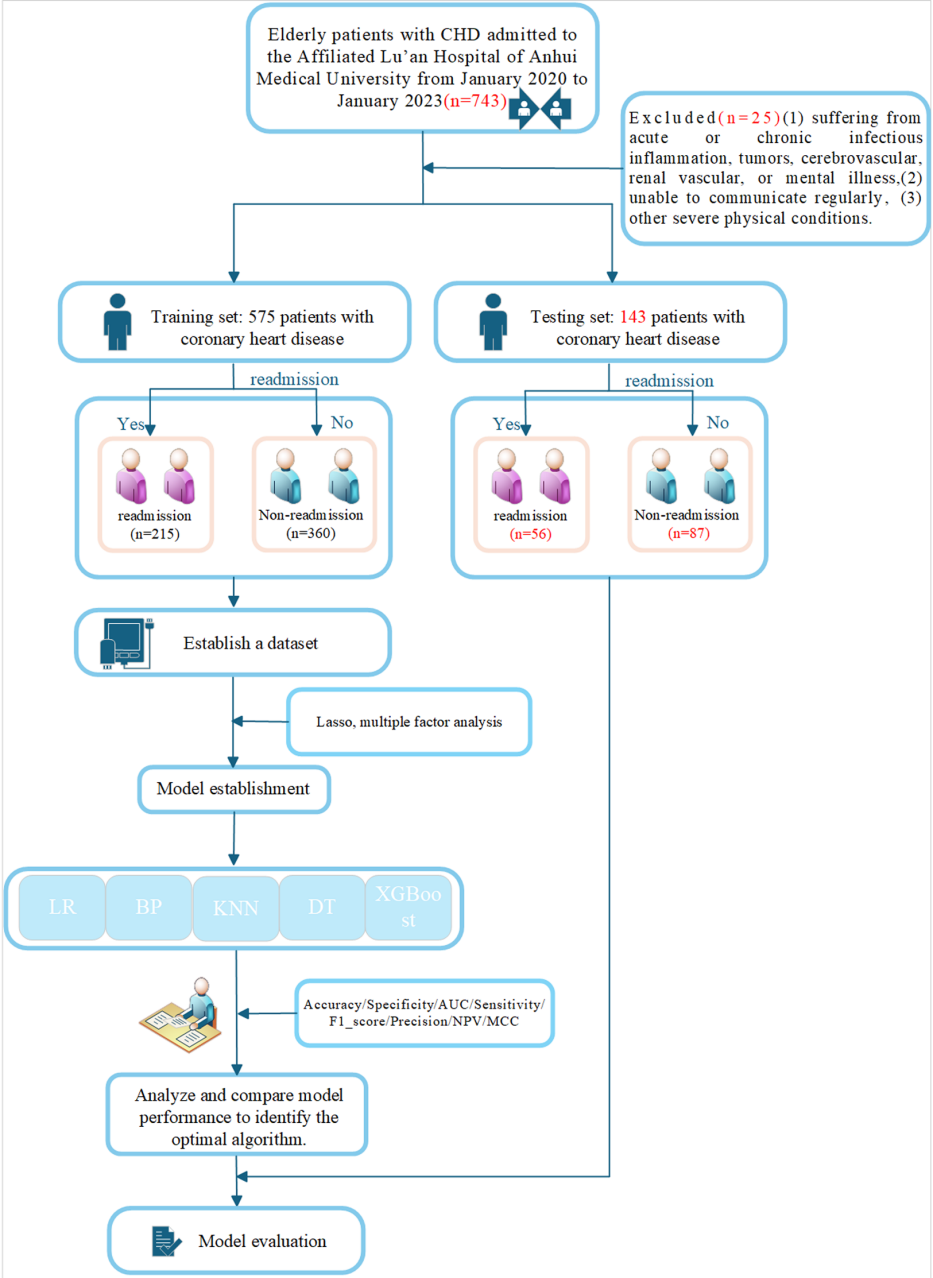
Flowchart.

Between January 2020 and January 2023, we identified 743 elderly individuals with CHD who were admitted to the Affiliated Lu'an Hospital of Anhui Medical University for our study. The following criteria had to be met in order to be included: (1) coronary angiography (CAG) showing coronary artery stenosis, (2) age ≥ 60, (3) readmission within 3 years, (4) complete clinical data. Criteria for exclusion: (1) Individuals suffering from acute or chronic infectious inflammation, tumors, cerebrovascular, renal vascular, or mental illness, (2) People unable to communicate regularly, (3) When combined with a tumor, secondary hypertension, or other severe physical conditions. This exclusion process resulted in a final study cohort of 718 patients after 25 were removed. The training set was composed of 575 of these elderly CHD patients, who were admitted to the same hospital during the aforementioned period. These patients were categorized into two groups based on their readmission status within three years: the readmission group, consisting of 215 patients, and the non-readmission group, with 360 patients. Concurrently, a validation set was established, which included 143 cases from a different affiliated hospital in Lu'an City. This validation set was further divided into a readmission group of 56 patients and a non-readmission group of 87 patients. The whole process of the study is shown in Figure 1. The study was conducted following the Declaration of Helsinki guidelines. The protocol was approved by the Ethics Committee of the Lu'an Municipal People's Hospital Affiliated to Anhui Medical University. Written informed consent was obtained from the patient. The ethical approval number of the study is 2023LLKS034.

### 2.2 Date preprocessing

A comprehensive dataset comprising 22 basic clinical data and laboratory-related tests of patients was collected: Sex, Height, Weight, body mass index (BMI), Age, Hypertension, Diabetes, history of smoking, Neutrophil, Monocyte, Lymphocyte, Red blood cell distribution width (RDW), MCV, Blood Glucose, Triglycerides (TG), High-density lipoprotein cholesterol (HDL), Low-density lipoprotein cholesterol (LDL), Creatine kinase isoenzyme MB (CKMB), Neutrophil-to -Lymphocyte ratio (NLR), Monocyte-to-HDL ratio (MHR), Monocyte-to-Lymphocyte ratio (MLR), TyG-BMI index, Triglyceride glucose-body mass index. Laboratory indices were determined using standard institutional laboratory measurements Lu “an Municipal People's Hospital Affiliated to Anhui Medical University. All measurements were carried out by personnel blinded to the patients” baseline characteristics and clinical outcomes.

The TyG-BMI index is an indicator that combines triglycerides, fasting blood glucose, and BMI to assess insulin resistance (IR). The TyG-BMl index was calculated as follows: BMI = weight (kg)/height (m^2^);

$$\mathrm{TyG}\;\mathrm{index}=\ln\;\left(\frac{\mathrm{fasting}\;\mathrm{triglyceride}\;(\mathrm{mg}/\mathrm{dL})\times \mathrm{fasting}\;\mathrm{glucose}\;(\mathrm{mg}/\mathrm{dL})}{2}\right)$$

TyG-BMI index = TyG index × BMI.

### 2.3 Machine learning algorithms

Although other machine learning methods excel in many situations, our research focuses on comparing traditional statistical methods with modern machine learning methods, and therefore logistic regression (LR), BP neural networks (BP), K Nearest Neighbor (KNN), extreme gradient boosting (XGBoost) and The Decision Tree (DT) have been chosen to represent them.

LR as a traditional statistical learning method, logistic regression is widely used in classification problems, especially in the fields of medicine and biostatistics. Its strength lies in the high interpretability of the model, which provides insights about the relationship between predictor variables and outcomes. In addition, logistic regression has relatively low data requirements and is suitable for handling the small to medium sized datasets in our study.

BP is a deep learning method that is capable of handling non-linear relationships and has strong generalization capabilities. We chose BP neural networks to explore more complex data patterns, especially when there are nonlinear relationships between variables.

KNN is an instance-based learning algorithm that is simple to implement and makes no assumptions about the distribution of the data, thus providing good classification in some cases.

XGBoost is an ensemble learning algorithm that builds on gradient boosting decision trees. It excels at handling large-scale datasets and is renowned for its strong generalization capabilities. XGBoost enhances model performance by fine-tuning the objective function through a gradient boosting approach. Additionally, it incorporates a regularization term that helps mitigate the risk of overfitting, making it a robust choice for various machine learning tasks.

DT is a straightforward and intuitive method for classification and regression. Its primary advantage lies in its high interpretability, which makes it easy to understand and visualize. By dividing data through a tree-like structure, Decision Trees effectively capture hierarchical relationships and interactions among features within the data. This clarity and simplicity make Decision Trees a popular choice for applications where model interpretability is crucial.

We understand that different algorithms have their unique advantages and applicable scenarios. In future research, we may consider incorporating other algorithms such as decision trees and random forests.

#### 2.3.1 XGBoost function (Sigmoid function)

The goal of XGBoost is to minimize an objective function consisting of a loss function worker and a regularization term Ω:

$$\mathrm{Obj}=\sum_{i=1}^{n}L(y_{i},\hat{y_{i}})+\sum_{k=1}^{K}\Omega (\;f_{k})$$

Here:

n is the number of samples, is the true value of the *i*-th sample, $\hat{y_{i}}$ is the predicted value of the *i*-th sample.

K is the number of weak learners (usually decision trees), $f_{k}$ representing the *k*-th weak learner.

$L(y_{i},\hat{y_{i}})$ is the loss function, such as the squared error $L(y,\hat{y})=(y-\hat{y}{)}^{2}$

$\Omega (\;f_{k})$ is the model's complexity penalty term, used to prevent overfitting.

**Figure 2:**
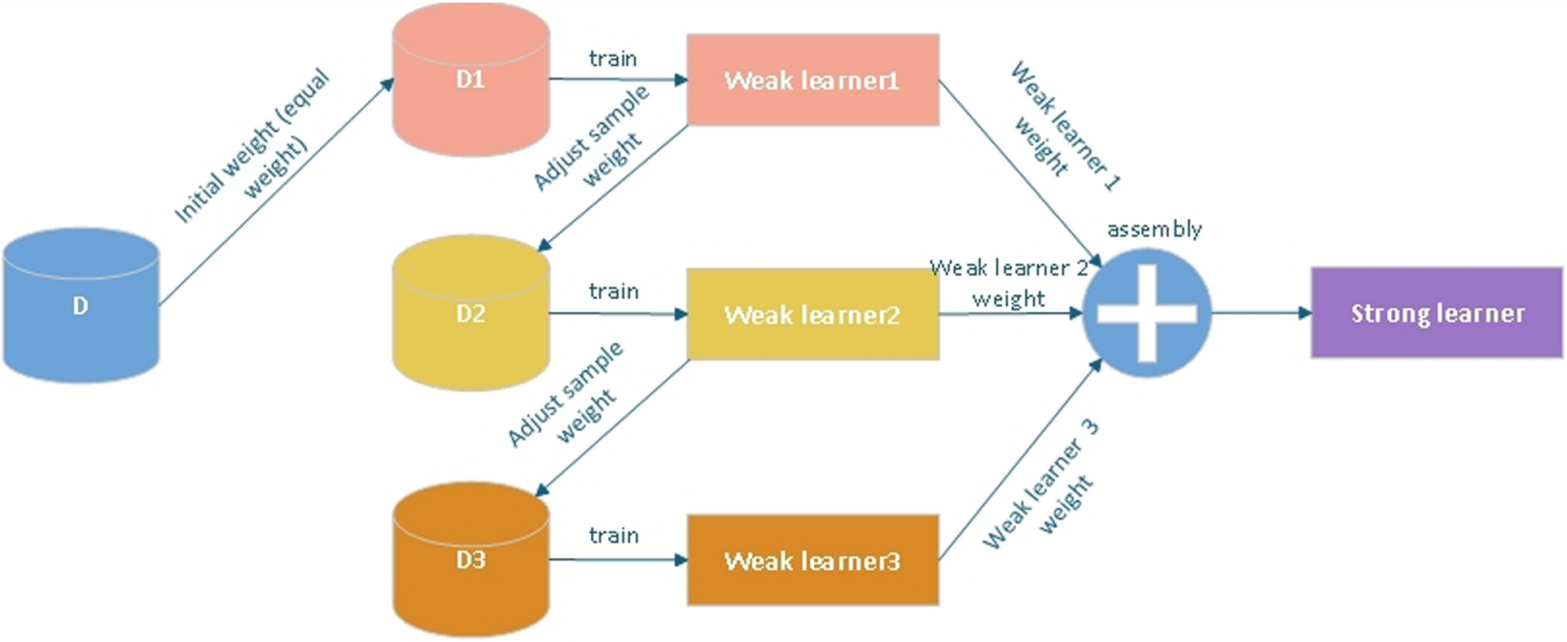
Basic principles of XGBoost model.

The core idea of XGBoost is to add a new weak learner (decision tree) in each round of training to minimize the residuals of the current model (Figure 2).

### 2.4 Evaluation standard

Classiffcation accuracy (ACC), Specificity, Sensitivity, Precision, F1_score, Negative Predictive Value (NPV), Matthews Correlation Coefficient (MCC) and the area under the operating characteristic curve (ROC) of the subject (AUC) were used to evaluate the model's quality.

The deffnition is shown below:

$$\mathrm{Accuracy}=\frac{\mathrm{TP}+\mathrm{TN}}{\mathrm{TP}+\mathrm{FP}+\mathrm{TN}+\mathrm{FN}}\times 100\text{\%}$$

$$\mathrm{Sensitivity}=\frac{\mathrm{TN}}{\mathrm{FP}+\mathrm{TN}}\times 100\text{\%}$$

$$\mathrm{Precision}=\frac{\mathrm{TP}}{\mathrm{TP}+\mathrm{FP}}\times 100\text{\%}$$

$$\mathrm{Specificity}=\frac{\mathrm{TN}}{\mathrm{FP}+\mathrm{TN}}\times 100\text{\%}$$

$$\mathrm{Recall}=\frac{\mathrm{TP}}{\mathrm{FP}+\mathrm{TP}}\times 100\text{\%}$$

$$\mathrm{Negative}\cdot \mathrm{predictive}\cdot \mathrm{value}=\frac{\mathrm{TN}}{\mathrm{TN}+\mathrm{FN}}\times 100\text{\%}$$

$$F1=\frac{2\;*\;\mathrm{Recall}\;*\;\mathrm{Precision}}{\mathrm{Recall}+\mathrm{Precision}}\times 100\text{\%}$$

for which TP, FN, TN, and FP reffect the number of true positives, false negatives, true negatives, and false positives, respectively.

### 2.5 Statistical methods

R software and Windows SPSS version 26.0 were utilized for the data analysis. Measurement data were expressed as mean ± standard deviation, median, and percentile, depending on whether they followed a normal distribution, while count data were expressed as percentage. The receiver operating characteristic (ROC) curve and calibration curve were used to assess the predictive performance of this column graph model in predicting readmission risk for elderly patients with coronary heart disease. The readmission risk prediction model for these patients was created using the R programming language. It was deemed statistically significant when *p* < 0.05.

## 3 Results

### 3.1 Baseline characteristics

The readmission group exhibited significant disparities in age, hematological parameters, glycemic levels, cardiac enzyme levels, and inflammatory markers when compared to the non-readmission group, which may imply a correlation with the risk of readmission. Conversely, variables such as gender, BMI, blood lipid profiles, and certain immune indicators did not demonstrate significant differences across both cohorts, suggesting that they might not be substantial predictors for readmission. The specific baseline data of the final training set and the test set are shown in Tables 1, 2.

**Table 1:** Baseline characteristics in training cohort.

Variables	Non-readmission group (360 cases)	Readmission group (215 cases)	*t*/*F*	*p*
Male, *n* (%)	79 (21.9)	137 (63.7)	0.071	0.789
Height (cm)	165.500 (160.000,170.000)	167.000 (160.000,170.000)	−0.804	0.422
Weight (kg)	68.000 (60.000,75.000)	70.000 (62.000,76.000)	−1.356	0.001
BMI (kg/m^2^)	24.563 (22.520,26.795)	24.974 (23.275,27.260)	−1.548	0.112
Age (years)	61.500 (55.000,69.000)	71.000 (64.500,76.000)	−9.982	0.001
Hypertension, *n* (%)				
Yes	84 (23.3)	132 (61.4)	0.115	0.735
No	276 (76.7)	83 (38.6)		
Diabetes, *n* (%)				
Yes	124 (34.4)	122 (56.7)	15.572	0.001
No	236 (65.6)	93 (43.3)		
Smoking, *n* (%)				
Yes	147 (40.8)	73 (34.0)	0.356	0.551
No	213 (59.2)	142 (66.0)		
Neutrophil (10^9^/L)	4.515 (3.445,6.340)	8.120 (6.200,9.935)	−13.129	0.001
Monocyte (10^9^/L)	0.470 (0.330,0.620)	0.460 (0.340,0.620)	−0.028	0.977
Lymphocyte (10^9^/L)	1.660 (1.208,2.173）	1.500 (1.100,1.990）	−2.781	0.005
MCV (fl)	89.900 (87.300,92.500)	91.700 (88.800,94.100)	−4.569	0.001
RDW-CV	12.700 (12.300,13.100)	13.300 (12.700,13.600)	−7.258	0.001
Blood glucose (mmol/L)	6.600 (5.900,7.500)	8.600 (7.000,10.200)	−10.925	0.001
TG (mmol/L)	1.410 (0.960,2.060)	1.390 (0.930,2.270)	−0.323	0.747
HDL (mmol/L)	1.200 (1.040,1.420)	1.220 (1.040,1.405)	−0.080	0.936
LDL (mmol/L)	2.890 (2.278,3.643)	2.940 (2.310,3.745)	−0.433	0.665
CKMB (ng/ml)	122.350 (72.030,409.750)	220.000 (87.000,1,071.000)	−3.841	0.001
NLR	2.686 (1.794,4.530)	5.311 (3.509,7.767)	−10.925	0.001
MHR	0.384 (0.258,0.534)	0.364 (0.264,0.558)	−0.124	0.902
MLR	1.418 (0.969,2.083)	1.438 (0.879,2.186)	−0.323	0.747
TyG-BMI index	37.648 (28.655,48.620)	48.683(38.301,59.686)	−7.362	0.001

Abbreviations: Data are presented as the number (%) or median (Q1, Q3). BMI, body mass index; RDW, red blood cell distribution width (RDW); TyG-BMI index, triglyceride glucose-body mass index; HDL, high-density lipoprotein cholesterol; LDL, low-density lipoprotein cholesterol; TC, total cholesterol; CKMB, Creatine kinase isoenzyme MB; NLR, Neutrophil-to -Lymphocyte ratio; MHR, Monocyte-to-HDL ratio; MLR, Monocyte-to-Lymphocyte ratio. *p* < 0.05 is considered statistically significant.

**Table 2:** Baseline characteristics in testing cohort.

Variables	Non-readmission group (87cases)	Readmission group (56 cases)	*t*/*F*	*p*
Male, *n* (%)	61 (70.1)	37 (66.1)	0.374	0.541
Height (cm)	166.000 (160.000,170.000)	165.000 (160.000,170.000)	−0.418	0.676
Weight (kg)	70.000 (62.000,76.000)	67.000 (60.000,75.000)	−0.740	0.460
BMI (kg/m^2^)	25.148 (23.125,27.102)	24.509 (22.857,26.913)	−0.438	0.661
Age (years)	60.000 (54.000,66.000)	70.000 (67.000,75.000)	−6.272	0.001
Hypertension, *n* (%)				
Yes	61 (70.1)	33 (58.9)	1.893	0.169
No	26 (29.9)	23 (41.1)		
Diabetes, *n* (%)				
Yes	19 (21.8)	38 (67.9)	30.097	0.001
No	68 (78.2)	18 (32.1)		
Smoking, *n* (%)				
Yes	30 (34.5)	21 (37.5)	0.135	0.713
No	57 (65.5)	35 (62.5)		
Neutrophil (10^9^/L)	4.090 (3.380,5.000)	6.260 (5.0500,7.330)	−5.172	0.001
Monocyte (10^9^/L)	0.460 (0.350,0.610)	0.510 (0.320,0.770)	−0.039	0.969
Lymphocyte (10^9^/L)	1.760 (1.210,2.130）	1.600 (1.040,2.000）	−1.425	0.154
MCV (fl)	92.500 (90.300,94.300)	93.900 (92.300,95.600)	−2.565	0.010
RDW-CV	12.600 (12.200,12.900)	13.500 (13.200,13.700)	−7.906	0.001
Blood glucose (mmol/L)	6.670 (5.910,6.500)	8.700 (6.5.00,9.300)	−10.925	0.001
TG (mmol/L)	1.410 (0.960,2.060)	1.390 (0.930,2.270)	−0.025	0.980
HDL (mmol/L)	1.200 (1.040,1.420)	1.220 (1.040,1.405)	−1.655	0.098
LDL (mmol/L)	2.890 (2.278,3.643)	2.940 (2.310,3.745)	−0.716	0.474
CKMB (ng/ml)	82.000 (68.000,106.000)	454.000 (284.000,1,030.000)	−9.246	0.001
NLR	2.347 (1.764,3.413)	3.813 (2.563,6.327)	−4.260	0.001
MHR	0.410 (0.269,0.478)	0.359 (0.205,0.520)	−0.813	0.416
MLR	0.264 (0.213,0.405)	0.300 (0.220,0.407)	−1.129	0.259
TyG-BMI index	37.648 (28.655,48.620)	48.683(38.301,59.686)	−7.362	0.001

Abbreviations: Data are presented as the number (%) or median (Q1, Q3). BMI, body mass index; RDW, red blood cell distribution width(RDW); TyG-BMI index, triglyceride glucose-body mass index; HDL, high-density lipoprotein cholesterol; LDL, low-density lipoprotein cholesterol; TC, total cholesterol; CKMB, Creatine kinase isoenzyme MB; NLR, Neutrophil-to -Lymphocyte ratio; MHR, Monocyte-to-HDL ratio; MLR, Monocyte-to-Lymphocyte ratio. *p* < 0.05 is considered statistically significant.

### 3.2 Feature selection

#### 3.2.1 Lasso regression analysis

**Figure 3:**
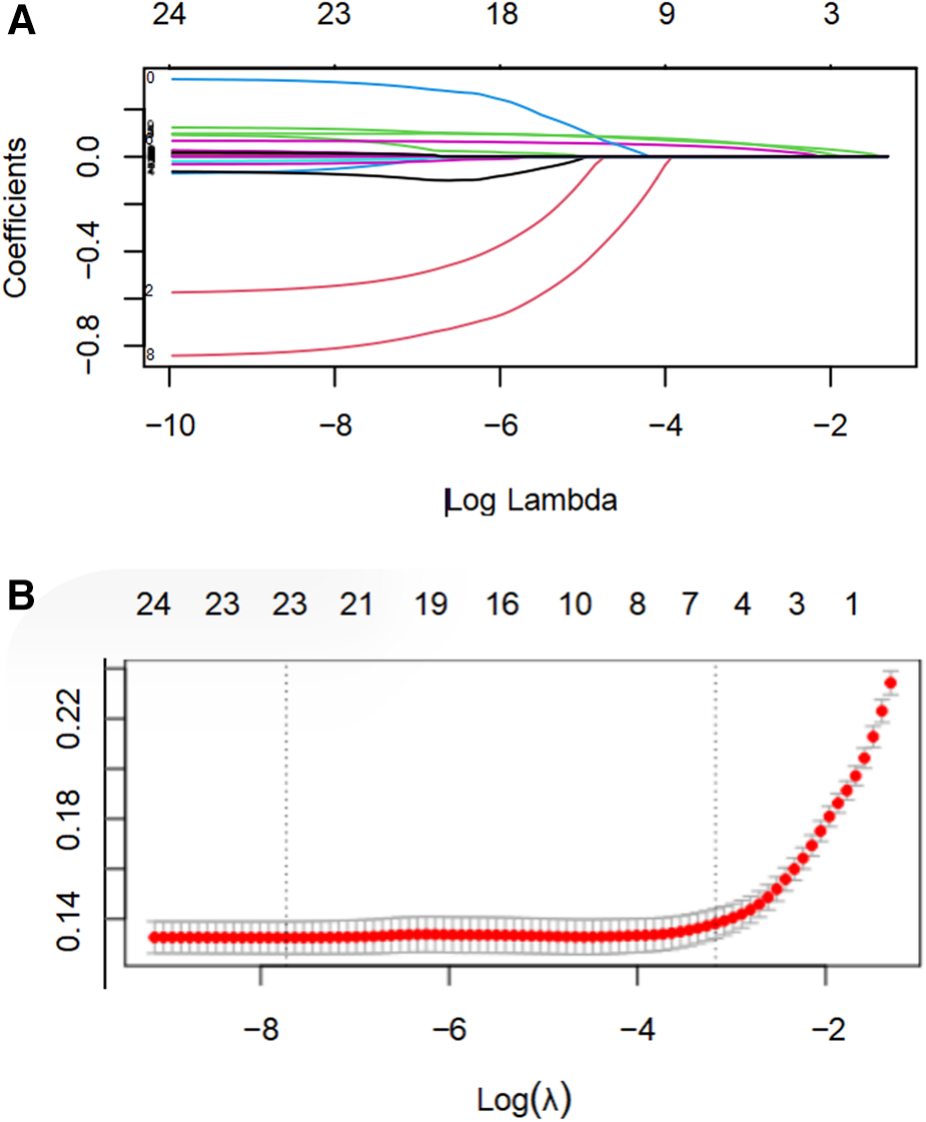
LASSO regression analysis was utilized to select characteristic factors. **(A)** Through 10-fold cross-validation, vertical lines were drawn at selected values, where the optimal lambda value resulted in six non-zero coefficients. **(B)** In the LASSO model, coefficient profiles of 22 texture features were plotted from the log(*λ*) sequence. The results indicated that at lg(*λ*) = 0.0419, the 22 independent variables were reduced to 6.

In Lasso regression analysis, as the value of *λ* increases, the coefficients of the variables gradually shrink towards zero. A higher *λ* applies a stronger penalty to models with more variables, resulting in a model with fewer selected features. The results indicated that at lg(*λ*) = 0.0419, the 22 independent variables were reduced to 6, including Age, blood glucose, TyG-BMI, RDW, CKMB, and diabetes, and they constitute significant predictors of readmission in older patients with CHD (Figure 3).

#### 3.2.2 Multivariate logistic regression

Lasso regression identified six candidate predictors: age, blood glucose, TyG-BMI, RDW, CKMB, and diabetes. Subsequently, these six features were subjected to multivariate logistic regression analysis. The findings show that among older patients with CHD, RDW, TyG-BMI, and diabetes are important variables predicting readmission (Table 3).

**Table 3:** Multivariate logistic regression analysis.

Variables	OR	95% CI (lower–upper)	*B*	*p*	Tolerance	VIF
Age	1.108	1.078–1.138	0.102	0.433	0.904	1.107
RDW	1.470	1.099–1.967	−0.821	<0.001	0.900	1.084
Blood glucose	1.766	1.540–2.025	−0.825	0.793	0.910	1.111
TyG-BMI	1.040	1.026–1.055	0.005	<0.001	0.965	1.036
CKMB	1.000	1.000–1.001	0.000	0.280	0.995	1.005
Diabetes	1.635	1.403–1.898	0.291	<0.001	0.948	1.054

Abbreviations: RDW, red blood cell distribution width (RDW); TyG-BMI index, triglyceride glucose-body mass index; CKMB, Creatine kinase isoenzyme MB; OR, odds ratio; CI, conﬁdence interval; VIF, variance inflation factor. *p* < 0.05 is considered statistically significant.

### 3.3 Evaluation and validation of models

**Figure 4:**
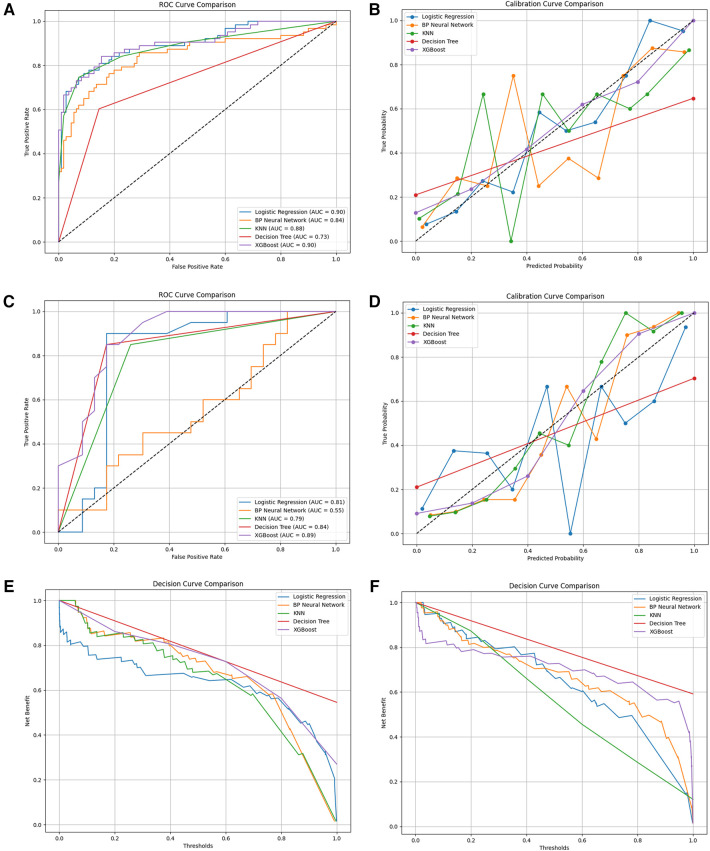
**(A)** the ROC curves for the prediction of readmission rates in the training set by five different models; **(B)** the calibration curves of the five models for predicting readmission rates in the training set; **(C)** the ROC curves for the prediction of readmission rates in the testing set by five different models; **(D)** the calibration curves of the five models for predicting readmission rates in the testing set; **(E)** the decision curves of the five models for predicting readmission rates in the training set; **(F)** the decision curves of the five models for predicting readmission rates in the testing set.

Machine learning models were developed using XGBoost, LR, RF, KNN and DT algorithms in combination with Selected variables to predict the unexpected readmission of elderly patients with coronary heart disease within three years in the training set. The predictive performance of the ML models was tested in the test set. (Table 4) lists the models' Accuracy, Specificity, AUC, Sensitivity and F1_score. The results indicated that XGBoost was the highest in the training set and testing set. Among these models, XGB demonstrated the highest AUC of 0.903 (Figure 4A). The calibration curve, a scatter plot of actual vs. envisioned incidence, is a crucial tool for assessing the model's prediction ability and a graphical representation of the Hosmer-Lemeshow goodness-of-fit test results. An ideal calibration curve will be near the 45-degree line, suggesting that the model's anticipated probabilities correspond with the actual probabilities. This helps us comprehend the relationship between the model's expected probability and the actual occurrence probabilities. Overall, XGBoost's performance is closer to the diagonal, suggesting that it has superior calibration. In contrast, the curves for decision trees and KNN exhibit greater fluctuations, likely due to these models' propensity to produce more extreme probability estimates (Figure 4B). An AUC of 0.891 was obtained through external validation (Figure 4C). The calibration curves of the XGB model also showed good calibration performance (Figure 4D). In the decision curve analysis across both training and test datasets, the decision tree and XGBoost models consistently exhibit superior net benefits within a broad spectrum of threshold values. This observation could imply that, within these particular threshold ranges, the predictive accuracy of these models substantially amplifies the efficacy of decision-making (Figures 4E,F).

**Table 4:** Comparison of the five prediction models.

	Model	LR	BP	KNN	DT	XGBoost
Training set	Accuracy	0.855	0.803	0.861	0.763	0.855
	Specificity	0.945	0.873	0.927	0.855	0.936
	AUC	0.900	0.843	0.884	0.729	0.903
	Sensitivity	0.697	0.683	0.746	0.603	0.714
	F1_score	0.778	0.717	0.797	0.650	0.783
	Precision	0.880	0.754	0.855	0.704	0.865
	NPV	0.846	0.828	0.864	0.790	0.851
	MCC	0.683	0.568	0.696	0.475	0.683
Testing set	Accuracy	0.558	0.488	0.791	0.837	0.837
	Specificity	0.826	0.522	0.739	0.826	0.826
	AUC	0.807	0.550	0.795	0.838	0.891
	Sensitivity	0.550	0.450	0.850	0.850	0.850
	F1_score	0.345	0.450	0.791	0.829	0.829
	Precision	0.556	0.450	0.739	0.810	0.810
	NPV	0.559	0.522	0.850	0.864	0.864
	MCC	0.550	0.485	0.589	0.675	0.675

Abbreviations: AUC, the area under the ROC curve; NPV, negative predictive value; MCC, Matthews Correlation Coefficient; LR, logistic regression (LR); BP, BP neural networks; KNN, K Nearest Neighbor (KNN); DT, the decision tree; XGBoost, extreme gradient boosting.

### 3.4 The SHAP to model interpretation

**Figure 5:**
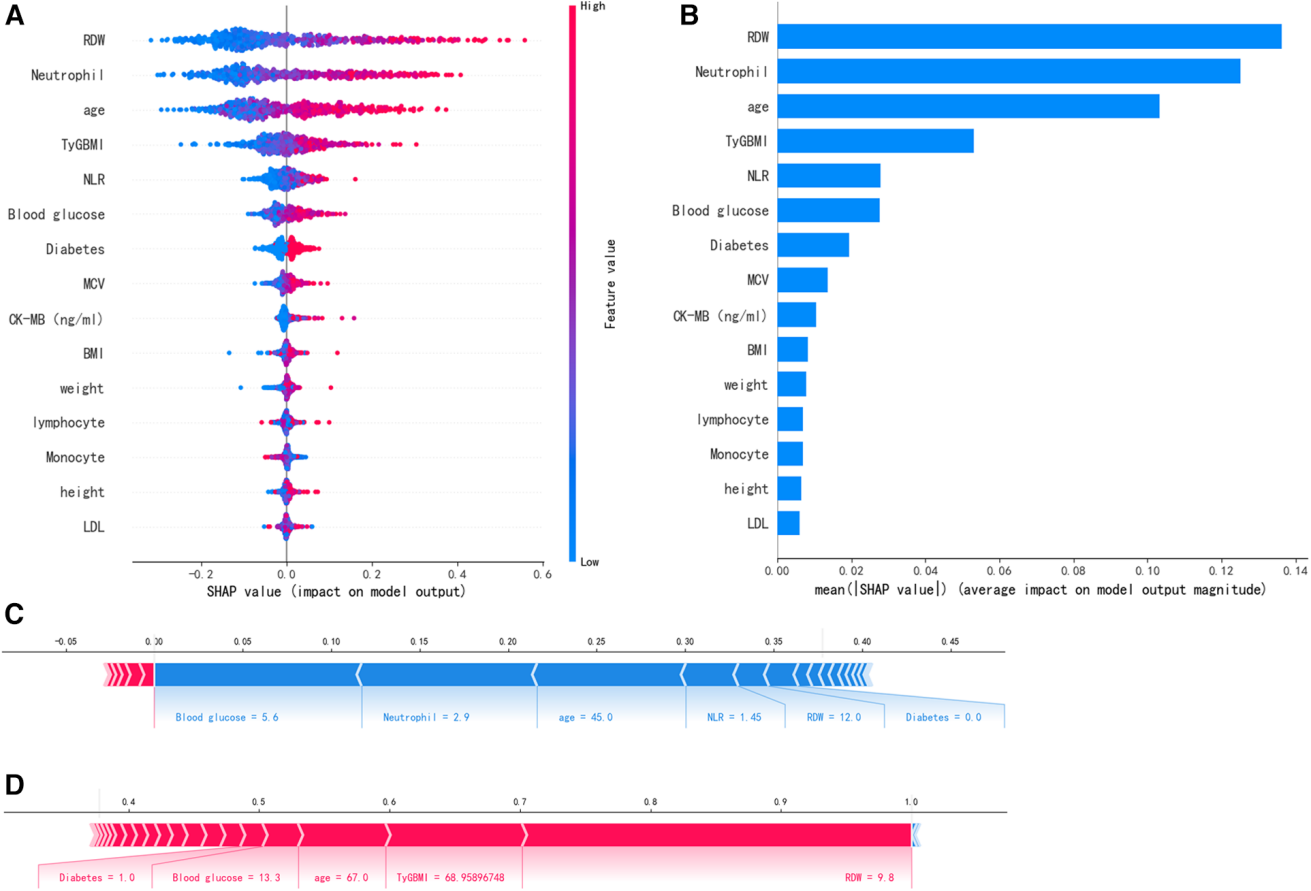
SHAP interprets the model. **(A)** Attributes of characteristics in SHAP. Each line represents a feature, and the abscissa is the SHAP value. Red dots represent higher eigenvalues and blue dots represent lower eigenvalues. **(B)** Feature importance ranking as indicated by SHAP. The matrix diagram describes the importance of each covariate in the development of the final prediction model. **(C)** Non-readmitted patients and **(D)** Readmitted patients.

To enhance the clinical applicability of the model, a compact XGBoost model was applied using the top 10 variables according to their mean absolute SHAP values, indicating their importance for prediction. SHAP values serve as a unified measure where a higher SHAP value for a feature indicates a higher risk of patient readmission. Figure 5A shows the fifteen most important features in our model. In each feature importance line, the contribution of all patients to the outcome is plotted with points of different colors, where red points denote high-risk values and blue points denote low-risk values. Upon admission, elevated levels of RDW, Neutrophil, age, TyG-BMI, NLR, blood glucose, and a history of diabetes are all factors that may heighten the risk of patient readmission. Figure 5B shows the ranking of nine risk factors evaluated by the average absolute SHAP value, with the *x*-axis SHAP value indicating the importance of the forecast model. Additionally, a new visualization method has been employed to make the results more intuitive. We provide two typical examples to illustrate the model's interpretability: one for a patient who was readmitted and one who was not. Arrows indicate the impact of each factor on the prediction, with blue and red arrows signifying whether the factor decreases (blue) or increases (red) the risk of death. The combined effect of all factors provides the final SHAP value, with a non-readmitted myocardial infarction patient having a low SHAP prediction score (0.00) (Figure 5C), while another readmitted patient has a higher SHAP score (1.00) (Figure 5D).

## 4 Discussion

### 4.1 High readmission rate among elderly patients with CHD

CHD is a form of cardiovascular ailment. In recent years, Its incidence has significantly increased in China, making cardiovascular diseases the leading cause of death for both urban and rural populations. Individuals afflicted with CHD must prioritize not only acute-stage treatment and rescue but also pre- and post-onset rehabilitation efforts. If patients fail to adhere to standard guidance, the likelihood of disease recurrence increases, potentially leading to repeated hospitalizations. This, in turn, exacerbates the medical burden, perpetuating a vicious cycle. With advancements in medical technology, coronary revascularization is increasingly employed in patients with acute coronary syndromes. This procedure rapidly alleviates narrowed or blocked coronary arteries, ameliorating symptoms of myocardial ischemia and enhancing patients' quality of life. Moreover, it facilitates their smoother reintegration into society, enabling them to fulfill their potential and contribute meaningfully to their communities (12). Percutaneous coronary intervention is a viable treatment option for narrow anomalies. However, patients may experience cardiac adverse effects following surgery, such as cardiogenic death or myocardial infarction, requiring re-hospitalization if risk factors for CHD continue.

### 4.2 The risk factors for readmission among elderly patients with CHD

The TyG-BMI is acknowledged as a useful predictor of cardiovascular illnesses and a dependable proxy measure for evaluating insulin resistance (IR) (13). Muniyappa R (14) has offered a deeper understanding of insulin's effects on vascular endothelium through the lens of mathematical modeling. The study highlights that under conditions of insulin resistance, the endothelium's sensitivity to insulin diminishes, resulting in impaired vasodilation. This reduction in nitric oxide (NO) availability is attributed to multiple factors, including a decrease in insulin-mediated NO synthesis and an increase in reactive oxygen species (ROS) generated during insulin resistance, which accelerates NO degradation. The imbalance of these vascular active substances contributes to the narrowing of blood vessels and fosters the progression of atherosclerosis. The TyG index is thought to be a more reliable measure of IR as it is generated from fasting triglycerides (FTG) and glucose levels. TyG-related metrics, including triglyceride-waist circumference (TyG-WC), triglyceride-waist-to-height ratio (TyG-WtHR) and TyG-BMI, have been shown to be particularly helpful in assessing IR, according to recent study (15). The TyG index was further combined with the BMI to consider the impact of obesity on heart disease risk. BMI is a measure of the ratio of weight to height and is used to assess whether an individual is overweight or obese. Among these, TyG-BMI has been associated with an increased risk of atherosclerosis and has been shown in several studies to have a predictive value for unfavorable cardiovascular events, namely fatal cardiovascular events (16, 17). In our study, the TyG-BMI index demonstrated greater predictive power in the group of elderly patients with coronary heart disease who were readmitted compared to those who were not. Serving as a composite marker of insulin resistance, the TyG-BMI index is notably correlated with the likelihood of readmission in this patient population. Insulin resistance is linked to vascular endothelial dysfunction, inflammation, and the advancement of atherosclerotic processes, which in turn can precipitate recurrent cardiovascular events and consequently raise the risk of hospital readmission. Thus, managing body weight and metabolic health could be instrumental in reducing the incidence of readmissions. Due to advances in genetic information, more academics are currently gaining an improved understanding of the etiological relationship between diabetes and CHD. Bhatti JS (18) has highlighted that in diabetic conditions, hyperglycemia promotes the generation of mitochondrial reactive oxygen species (ROS), increases the formation of advanced glycation end products (AGEs) within cells, activates protein kinase C (PKC), and enhances the flux through the polyol pathway. ROS directly increase the expression of inflammatory and adhesion molecules, contribute to the formation of oxidized low-density lipoproteins, and lead to insulin resistance. They activate the ubiquitin pathway, inhibit the activation of AMP-activated protein kinase (AMPK) and adiponectin, and reduce the activity of endothelial nitric oxide synthase (eNOS), all of which accelerate the development of atherosclerosis, leading to the progression of diabetic vascular complications. Goodarzi MO (19) and colleagues further substantiated the causal link between type 2 diabetes and coronary heart disease through Mendelian randomization studies. Subsequently, they exploited this genetic link to guide treatment plans for both diseases. This result is consistent with the current study's findings. Previous studies (20) have proved that good control of blood sugar can effectively reduce the incidence of diabetic microvascular complications. Our study's findings further confirm the correlation between diabetes and the rate of hospital readmissions. Patients with diabetes often experience chronic hyperglycemia, which can accelerate the progression of atherosclerosis and increase the risk of cardiovascular events. Moreover, individuals with diabetes may require more frequent medical interventions, including pharmacological treatments and surgeries, potentially raising the likelihood of readmission. Therefore, optimizing the management of diabetes, encompassing blood glucose control and the management of cardiovascular risk factors, is crucial for reducing the rate of readmissions. RDW holds significance in blood tests as it objectively indicates variations in the size of red blood cells. The insights gleaned from this parameter are pivotal for evaluating and predicting the risk of certain diseases. Elevated RDW levels have been proven to be significantly linked with both mortality and the advancement of cardiovascular illnesses in a number of studies (21–23). In patients suffering from chronic heart failure, there is a significant correlation between the RDW and poor prognosis. With the deterioration of heart function and the increase in hospital readmissions, RDW exhibits a trend of gradual increase, which is markedly correlated with a higher mortality rate. Notably, the RDW levels in heart failure patients are significantly elevated compared to the control group, and they rise notably with the advancement of the New York Heart Association (NYHA) functional classification. Research by Poz D et al. (24) shown the clinical usefulness of standard blood test indices, including RDW, when making preliminary diagnoses of coronary heart disease patients' symptoms. The substantial association between RDW and the severity of coronary heart disease was also noted by Wang H et al. (25). The results of this study provide insightful recommendations for clinical diagnosis and therapy. Our research findings demonstrate a significant correlation between RDW and the readmission rates among elderly patients with coronary heart disease. The increase in RDW is likely associated with inflammation, oxidative stress, and vascular damage, factors that could potentially elevate the risk of cardiovascular events, consequently leading to readmissions. Consequently, RDW may act as an early warning sign, assisting in the identification of high-risk patients who necessitate more intensive surveillance and intervention. In conclusion, our study highlights the significance of the TyG-BMI index, RDW, and diabetes in the context of readmission risks for elderly patients with coronary heart disease. These discoveries pave the way for future research directions, especially regarding how to mitigate readmission rates by optimizing these indicators. Subsequent studies can delve deeper into the causal relationships between these markers and readmission rates, and evaluate the efficacy of intervention strategies, aiming to provide more informed guidance for clinical practice.

Previous studies assessed all-cause readmissions 30 days or 1 year after cardiovascular events. Okere et al. (26) employed the decision tree algorithm to forecast the 30-day readmission rate among 346,390 hospitalized patients (aged ≥40 years), primarily diagnosed with ischemic heart disease. The model performed well, with all metrics exceeding 0.95, including accuracy, precision, recall rate, and area under the curve (AUC). Nevertheless, the calibration capacity of the model was not assessed in this work. A prediction model for readmission within 30 days was developed by Gupta et al. (27) using six machine learning algorithms. However, the model's best C statistic was only 0.641. With an AUC ranging of 0.681 to 0.720, Chinese researchers created nine machine learning models to forecast the likelihood of 30-day unplanned all-cause readmission (28). The research study by Forrest IS et al. (29) developed and validated a machine learning-based predictive model for coronary artery disease. The model used 95,935 electronic health records to assess the probability of coronary artery disease as a virtual score for coronary artery disease [ranging from 0 (lowest probability) to 1 (highest probability)]. The results showed that the model was able to predict coronary artery disease with an AUC of 0.95 and AUCs of 0.93 and 0.91 in the BioMe validation set and retention set, respectively. In addition, ISCAD scores were significantly associated with coronary artery stenosis, obstructive coronary artery disease, multibranched coronary artery disease, all-cause mortality, and coronary artery disease sequelae in terms of Clinical outcomes were significantly associated. A study by Huang AA and Huang SY (30) explored the use of machine learning in identifying risk factors for coronary artery disease. The researchers used various machine learning algorithms to analyze data to identify potential risk factors associated with coronary artery disease. This study by Saeedbakhsh S et al. (31) was based on machine learning algorithms (support vector machines, artificial neural networks, and random forests) to diagnose coronary artery disease. The study compares the performance of these algorithms in diagnosing coronary artery disease, providing valuable insights into clinical diagnosis. By introducing these studies, we are able to discuss more comprehensively the application of machine learning in cardiovascular disease research and provide a solid scientific foundation for our research. In this study, we have crafted an XGBoost algorithm-based predictive model tailored for forecasting the risk of readmission among elderly patients with coronary heart disease. Our model exhibits distinct strengths and a few limitations. Notably, the XGBoost model achieved the highest AUC scores in both the training and testing datasets (0.903 and 0.891, respectively), demonstrating superior predictive accuracy that outperforms many conventional statistical approaches and other machine learning models. Additionally, by employing Lasso regression and multivariate logistic regression, our model successfully identified critical predictive factors, including the TyG-BMI index, RDW, and diabetes, offering tangible targets for clinical intervention. Moreover, the use of SHAP values allows our model to elucidate the influence of each feature on the prediction outcome, thereby enhancing its interpretability and clinical utility. However, our model is not without its constraints. The sample, originating from a specific region in China, may harbor environmental and demographic biases that could potentially limit the model's generalizability to other regions or countries. Our research considered only 22 clinical and laboratory indicators, potentially overlooking other valuable predictive factors, which might restrict the model's predictive capacity. Although external validation was conducted on a dataset from a different hospital, the relatively small sample size (143 cases) could affect the robustness of the validation findings. In comparison with existing CHD risk prediction models, our approach diverges in terms of algorithm selection and feature engineering. While some existing models may depend on traditional statistical methods such as the Cox proportional hazards model, our model leverages advanced machine learning techniques, particularly XGBoost, which excels in managing imbalanced data and enhancing predictive precision. Our model is specifically designed for the elderly population with coronary heart disease, in contrast to many existing models that cater to a broader demographic. In summary, our XGBoost model has proven to be highly accurate and practical for predicting the readmission risk in elderly coronary heart disease patients. Future studies should aim to validate the model across a more diverse population and consider including additional environmental and lifestyle factors to bolster the model's generalizability and applicability.

The utilization of deep learning in heart disease prediction models and how deep learning can enhance model generalization and predictive accuracy can be further investigated in future projects. As wearables and technology advance, deep learning models could be applied to real-time patient health monitoring and readmission risk prediction. Better integration with clinical processes will be necessary for the future development of deep learning models in order to facilitate the use of these tools by doctors in their clinical practice. Deep learning in coronary readmission models has a bright future overall, but there are still a number of obstacles to be addressed, including societal, ethical, and technical ones. Future developments and breakthroughs are anticipated to continue as technology develops and research intensifies.

## 5 Conclusion

TyG-BMI, RDW, and diabetes mellitus at the time of admission are the factors affecting readmission of elderly patients with CHD. The readmission risk prediction model, constructed using the XGBoost algorithm, demonstrates commendable predictive efficacy. This model serves as a valuable tool for identifying high-risk individuals and implementing timely intervention strategies.

### 5.1 Limitation

Meanwhile, this study is subject to certain limitations. Initially, during the study's design phase, our selection criteria for variables focused mainly on the laboratory tests and patient histories obtained at admission, yet we neglected to incorporate assessments like the Gensini score or GRACE risk score, which are pivotal for gauging the severity of coronary artery disease and forecasting patient outcomes. Moreover, we overlooked the impact of interventional procedures and medication regimens on readmission risks, factors that are known to significantly influence the likelihood of patients being readmitted. Consequently, our model may have missed capturing some critical elements that affect the risk of readmission. To bolster the predictive accuracy of our model, it is imperative that future studies take into account a more holistic set of potential factors that could influence the risk of readmission. Additionally, the fact that this was a retrospective study conducted at a single center made it susceptible to selection bias and resulted in a paucity of long-term follow-up information on clinical outcomes and quality of life. Therefore, Prospective, multicenter studies are warranted for further validation and refinement of the predictive model. Additionally, larger sample sizes and inclusion of diverse populations would enhance model accuracy.

## Data Availability

The original contributions presented in the study are included in the article/Supplementary Material, further inquiries can be directed to the corresponding author.
